# Change of gait after unilateral vestibular neuritis: a prospective longitudinal observation study

**DOI:** 10.1038/s41598-021-00665-0

**Published:** 2021-11-03

**Authors:** Sung-won Chae, Jae-Jun Song, Woo-Sub Kim

**Affiliations:** 1 Department of Otorhinolaryngology-Head and Neck Surgery, Korea University Guro Hospital, Seoul, Republic of Korea; 2 Department of Physical Medicine & Rehabilitation, Eulji University Uijeongbu Hospital, Dongil-ro, 712 Uijeongbu-si, Gyeonggi-do, Republic of Korea

**Keywords:** Diseases, Health care, Medical research, Neurology, Signs and symptoms

## Abstract

Although symptoms of unilateral vestibular neuritis (uVN) resolve spontaneously within several weeks, recovery of gait function has unclearness in gait parameter changes and mediolateral stability improvements. In addition, prospective longitudinal studies on gait parameters after uVN are lacking. This study was conducted to reveal longitudinal change of gait function after acute uVN and to help the precise rehabilitation planning. Twenty three participants with uVN and 20 controls were included. 3D gait analyses were conducted three times after uVN onset at monthly intervals. From the gait analysis data, spatio-temporal parameters, inclination angle (IA) representing the relationship between center of mass (CoM) and center of pressure (CoP) in the frontal plane, and IA variability were obtained. Time effects on gait metrics were tested. Walking speed of participants with uVN improved significantly between the 1st and 3rd tests, but they were not significantly different to that of control, even in the 1st test. The step width of participants with uVN was significantly larger than that of control in the 1st test and improved significantly in the 2nd test. Variability of IA in affected side was significantly larger than that in controls in the 1st test and improved significantly in the 3rd test compared to the 1st test. Improvement of overall gait function and mediolateral stability during gait continued after acute stage of uVN (two months from onset in this study). Rehabilitation intervention should be continued after the acute stage of uVN to enhance appropriate adaptation in gait.

## Introduction

The acute stage of unilateral vestibular neuritis (uVN) lasts a few days to several weeks and has drastic consequences on balance control^1,2^. After the acute stage of uVN, there is a recovery of balance function by neuronal and behavioral plasticity^2,3^. The recovery of balance function occurs over weeks and months through the peripheral recovery and the process of central compensation: restoration, habituation, and adaptation.

Gait is an essential body function for activities of daily living. During acute stage, individuals with uVN have deterioration in gait function because the vestibular dysfunction elicit static and dynamic deficit in posture and balance control during gait^2,4,5^. After acute stage, the dynamic deficit of vestibular function considerably persists while static deficit almost disappears^6^. Patients with uVN can walk within 48 h after onset and can return to normal activities in about two weeks^6^. However, some cases show persistent postural imbalance and gait deviation. Because gait is a complex body function related with vestibulo ocular reflex (VOR), vestibular spinal reflex (VS), reticulo spinal reflex, cerebellum and higher cortical functions, severity of vestibular dysfunction has limitations in predicting gait disturbances after uVN. Although dysfunction of the VOR is an underpinning of symptoms in uVN, there is conflicting evidence regarding the relationship between VOR recovery and gait function^5^. Weak correlations between VOR and gait function suggest that gait function should be evaluated independently of VOR recovery^7^.

Gait dysfunction after uVN has been frequently assessed using clinical scales such as the Berg Balance Scale, Timed Up and Go, and Activities-specific Balance Confidence Scale. Although clinical scales are easy to use, quick to perform, and inexpensive, they are subjective, have ceiling effects, are not responsive to small changes, and do not reflect the underlying mechanism of balance control^8^. Therefore, to detect precise changes in gait after uVN, it is necessary to assess gait function by quantitative parameters from instrumented gait analysis systems. Previous studies have reported differences in spatio-temporal parameters between vestibular disorders and healthy controls^9,10^. Although longitudinal change of gait function after acute uVN is helpful for rehabilitation planning, it is unclear due to lack of reports. Although spatio-temporal parameters are comprehensive and basic outputs from instrumented gait analysis system, they have limitations in providing direct evidence for the biomechanical mechanism of balance control during gait.

Dynamic balance during gait can be assessed by the relationship between the center of mass (CoM) and center of pressure (CoP), which provides information relevant to the biomechanical mechanism of balance control during gait^8^. Unilateral vestibular dysfunctions usually show postural sway and a tendency to fall toward the affected side during gait, which suggests a more lateral displacement of CoM toward the CoP of the affected side. However, there is no report on whether the CoM displaces closer to the CoP of the affected side in the frontal plane after the onset of uVN compared to healthy controls. Although postural sway improves after the acute stage of uVN, the extent and timing of improvement of sway are still unclear. Allum et al. reported that postural sway reached a normal range at 3 months^11^*.* Halmagyi et al. postulated that increased body sway during walking remained for approximately 3 months^6^. These contrary findings suggest unclearness in temporal profile of the CoM-CoP relationship recovery in the frontal plane. Balance control during gait, especially in the frontal plane, is related to increased fall risk in individuals with balance problems^12^. Therefore, revealing the characteristics of the CoM-CoP relationship after uVN is clinically important to accurately determine the remaining gait dysfunction and to appropriately apply vestibular rehabilitation.

This study aimed to investigate the recovery of gait function after the onset of uVN. We tested the time effects on spatio-temporal parameters and the CoM-CoP relationship and explored differences in gait metrics between uVN and controls.

## Methods

### Participants

This study was a prospective study conducted at the Korea University Guro Medical Center. This study was conducted in accordance with the Declaration of Helsinki. Informed consent was obtained from all participants, and authorization and continued monitoring of the study protocol was obtained from the Korea University Guro Medical Center Institutional Review Board (approval number 2017GR0073). Participants with acute vertigo were referred for 3D gait analysis from January 2018 to January 2019 and participants with uVN were included in the data analysis. Diagnosis of uVN was confirmed by an otology specialist through medical history, physical examination, and clinical tests, including a bithermal caloric test (Jongkee’s formula)^13^. Canal paresis was defined as a response difference of more than 25% between the ears. After diagnosis, education of vestibular ocular rehabilitation exercise was provided for recovery. Exercises involved simple gaze stabilization that elicited the VOR (i.e., head tunning in the horizontal plane for 1 min while staring at a finger held at eye level and repeated in the vertical plane). The exercises were performed first sitting and then standing, four to five times per day by patient themselves. Exclusion criteria of participants in the data analysis were as follows: (1) Meniere disease, recurrent vestibulopathy, or benign-paroxysmal-positional-vertigo; (2) comorbidities in the central nervous system, such as cerebral infarction; and (3) medical history of musculoskeletal problems that could disturb normal walking, such as joint contracture and severe peripheral neuropathy. Participants in the control group were recruited from the physical medicine and rehabilitation department and did not have neuromuscular disease disturbing gait, confirmed by one physiatrist.

### Measurements

The perception and severity of dizziness were measured using the Dizziness Handicap Inventory (DHI) at the first visit, and after 4 weeks and 8 weeks. The DHI is a 25-item self-report questionnaire that assesses the impact of dizziness on daily life^14,15^. Computerized dynamic posturography (CDP) (Smar Balance Master, NeuroCom International Inc., Portland, OR, USA) was conducted at 1st visit, and after 4 weeks and 8 weeks. From the sensory organization test of CDP, the vestibular score and composite score were obtained^16,17^.

**Figure 1:**
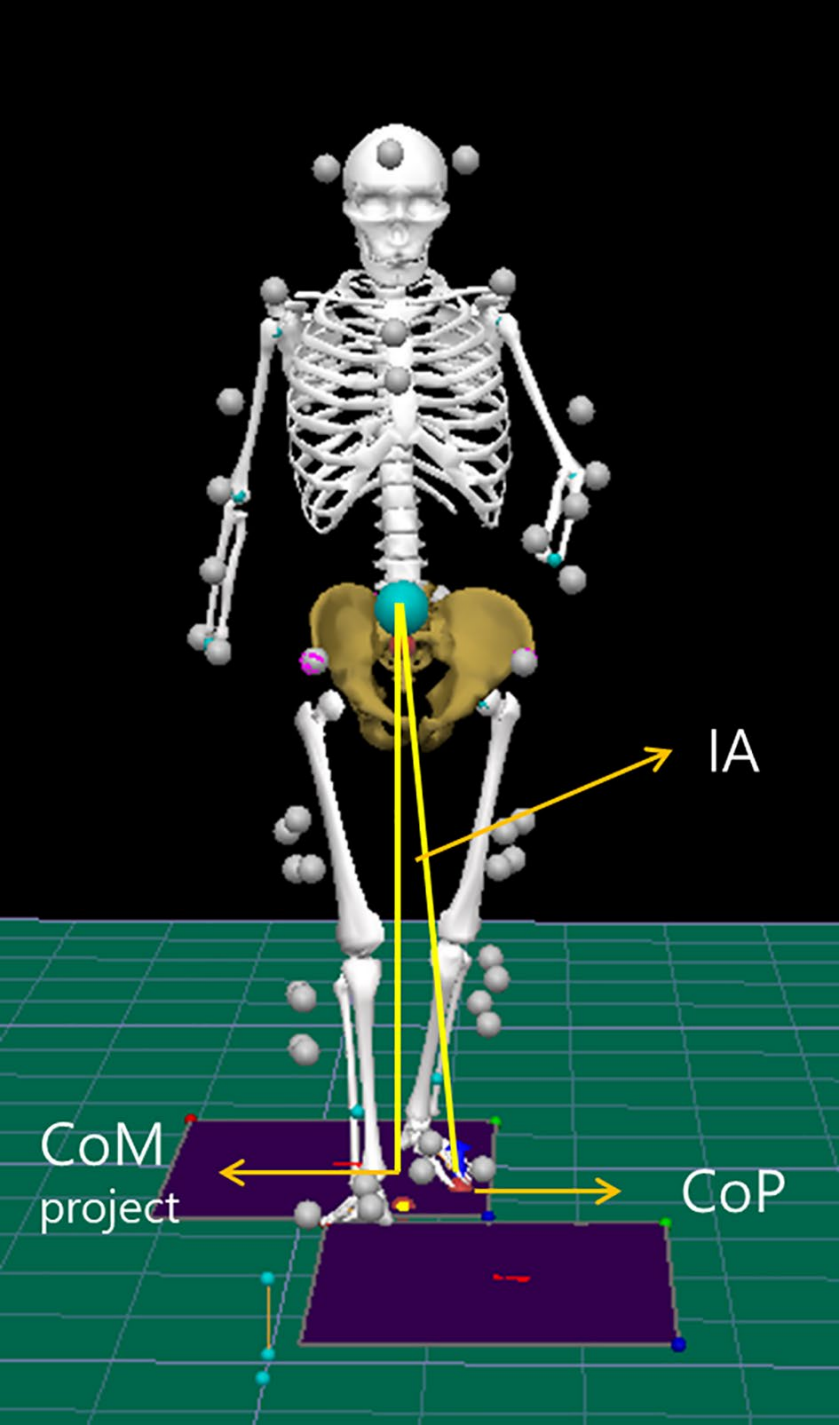
Fifty-six reflective markers were attached to the head, trunk, pelvis, arm, forearm, thigh, leg, and foot segments. IA was obtained with CoM, CoM projection on XY plane, and CoP. Vector1 from CoM to CoP projection, and vector2 from CoM to CoP were obtained. The angle between vector1 and vector2 was obtained with the right-hand rule by Visual3D. The angle obtained was projected to the YZ plane (sagittal) and XZ plane (frontal). When CoM did not go lateral to the CoP in the frontal plane, the left limb IA was the positive sign and the right limb IA was the negative sign. Therefore, we changed the right frontal plane IA sign by multiplication of (− 1). This image is obtained from Visual3D (ver6.2). *IA* inclination angle, *CoM* center of mass, *CoP* center of pressure.

3D motion analysis for level walking was conducted within two weeks of the initial visit to the otology clinic when participants could walk independently. Motion analyses were repeated after 4 and 8 weeks. 3D motion analysis for controls were conducted without repetition after 4 and 8 weeks. The motion analysis laboratory had an 8-m walkway and force plates embedded in the middle of the walkway. The ground reaction forces were measured using two force plates (Kistler, Type 5233A, Switzerland) with a frequency of 1200 Hz. An optoelectronic motion analysis system (Qualisys, Qualisys Medical AB, Gothenburg, Sweden) with eight cameras (Oqus 500 + , Qualisys Medical AB, Gothenburg, Sweden) was used to capture 3D trajectories of reflective markers at 120 Hz, which were filtered with Butterworth low pass filter with 6 Hz. Fifty-six reflective markers were attached to the head, trunk, pelvis, arm, forearm, thigh, leg, and foot segments recommended by Visual3D (C-motion Inc., Rockville, Maryland, USA) (Fig. 1). The explicit target was set parallel to the laboratory anterior–posterior axis to inform the target direction for participants during the walking trials. Participants performed steady state walking at a self-selected speed. To ensure safety issues, the development of discomfort or fatigue was checked, and walking tests were stopped when the patients complained of fatigue or discomfort. More than three trials that had clear contact on the force plate were obtained.

### Gait data analysis

Visual3D software was used to calculate the temporospatial, kinematic, and kinetic parameters. Joint angles were computed relative to the proximal segment. The head and pelvis angles were the segment angles with respect to the global coordinate reference. Joint movement and power were calculated in the hip, knee, and ankle. CoP during the stance phase was calculated from the force plate data for each side. CoM data were provided by Visual3D, which calculated CoM from the kinematics of segments and anthropometric data. The CoM-CoP inclination angle (IA) was calculated in the frontal plane of the global coordinate system (Fig. 1). IA on the affected side (IA_aff) and IA on the non-affected side (IA_nonaff) were determined according to the side of uVN. IA was observed during the stance phase. A minimum value of IA (IA_min) was obtained in each lower limb stance phase. Variability of IA (IA_var) was calculated with normalized root mean squared error^18–20^ in each participant. Gait metrics from controls were the averages of both sides.

### Statistics

Descriptive statistics for age, sex, and lesion side were conducted. Data normality was investigated with histogram and Shapiro–Wilk test. Independent sample t-test and chi-square test were used to compare the uVN and control groups. A linear mixed model (LMM) was used to investigate the effect of time on DHI, vestibular score, composite score, and gait metrics in uVN. The LMM allows estimation of the effects of explanatory variables, fixed effects, including time and side while statistically controlling the effects of participants, and random effects. This approach was implemented to investigate whether there was a significant change in the dependent variables across time (1st, 2nd, and 3rd test) and side (affected vs. non-affected). Fixed effect “side” was included when dependent variables were measured separately in the affected and non-affected sides. Multiple models were run, and the likelihood-ratio test was used to investigate if introducing fixed effects fitted with maximum likelihood while keeping the random effect the same. Therefore, the likelihood-ratio test via ANOVA was used to compare the goodness of fit of different models. R 3.32 statistical software (R Foundation for Statistical Computing, 2016) was used for all statistical analyses. All models were fitted using the “lmer” function in R. R package “lmerTest” was used to compute least-squares means and pairwise differences of these and P-values (Kuznetsova et al., 2017). The required minimum sample size was 20 cases which were calculated with a 5% significance level, 80% power, effect size 0.3, and three repetitions within factors by G*Power 3.1.9.2 software. Statistical significance was set at P < 0.05.

### Ethics approval and consent to participate

Informed consent was obtained from all participants, and authorization and continued monitoring of the study protocol was obtained from the Korea University Guro Medical Center Institutional Review Board.

## Results

### General characteristics of participants

Of the 122 participants referred for the 3D gait analysis study, 27 participants were confirmed with uVN. Of the 27 participants, four did not participate in the follow-up test. Hence, 23 participants with uVN were included in the data analysis. The control group included 20 healthy adults. The general characteristics of the participants are described in Table 1. There were no significant differences in age, height, weight, and sex ratio between the uVN and control groups.

**Table 1:** Comparison of general characteristics between uVN and control groups.

	uVN (n = 23)	Control (n = 20)	P-value
Age (years)	57.57 (11.60)	57.10 (9.64)	0.89
Height (m)	1.61 (0.09)	1.62 (0.08)	0.66
Weight (kg)	64.5 (14.52)	64.2 (11.3)	0.94
Sex (female/male)	13/10	9/11	0.65
Side of uVN (left/right)	7/16		
Acute onset duration (days)	4.6 (3.3)		

Comparisons between uVN and control were conducted using the t-test and chi-square test. Values are presented as mean (standard deviations).

*uVN* unilateral vestibular neuritis.

### Changes in DHI, vestibular score, and composite scores

The results of the LMM analysis with the likelihood-ratio test to compare the goodness of fit of different models are reported in Table 2. The time effect had a significant fixed effect on DHI (chisq = 24.89, P < 0.01), vestibular score (chisq = 8.22, P = 0.02), and composite score (chisq = 10.28, P < 0.01). Post-hoc analysis showed significant differences between the 1st and the other tests (2nd and 3rd) in DHI, vestibular score, and composite score (Table 3).

**Table 2:** Results from likelihood-ration test via ANOVA to test the significance of time and side effects.

**A. Model 1: DV = 1 + (1|ID), Model 2: DV = 1 + time + (1|ID)**								
DV = DHI	df	AIC	BIC	logLik	Deviance	Chisq	Df	P
Model 1	3	615.61	622.31	− 304.81	609.61			
Model 2	5	594.74	605.91	− 292.37	584.74	24.868	2	** < 0.01**
DV = vestibular score								
Model 1	3	625.17	631.88	− 309.59	619.17			
Model 2	5	620.96	632.13	− 305.48	610.96	8.2174	2	**0.02**
DV = composite score								
Model 1	3	517.09	523.79	− 255.54	511.09			
Model 2	5	510.81	521.98	− 250.41	500.81	10.276	2	** < 0.01**
DV = speed								
Model 1	3	− 80.387	− 73.685	43.194	− 86.387			
Model 2	5	− 83.177	− 72.007	46.589	− 93.177	6.7901	2	**0.03**
DV = stride length								
Model 1	3	− 109.89	− 103.19	57.947	− 115.89			
Model 2	5	− 113.00	− 101.83	61.502	− 123.00	7.1112	2	**0.03**
DV = cadence								
Model 1	3	494.56	501.26	− 244.28	488.56			
Model 2	5	497.92	509.09	− 243.96	487.92	0.6387	2	0.73
DV = step width								
Model 1	3	− 310.24	− 303.54	158.12	− 316.24			
Model 2	5	− 312.50	− 301.33	161.25	− 322.50	6.2541	2	**0.04**

**B. Model 1: DV = 1 + (1|ID), Model 2: DV = 1 + time + (1|ID), Model 3: DV = time × side + (1|ID)**								
DV = step length	df	AIC	BIC	logLik	Deviance	Chisq	Df	P
Model 1	3	− 449.51	− 440.73	227.75	− 455.51			
Model 2	5	− 455.63	− 441.00	232.82	− 465.63	10.125	2	** < 0.01**
Model 2	5	− 455.63	− 441.00	232.82	− 465.63			
Model 3	8	− 450.60	− 427.19	233.30	− 466.60	0.9716	3	0.81
DV = stance phase								
Model 1	3	− 719.39	− 710.61	362.69	− 725.39			
Model 2	5	− 722.73	− 708.09	366.36	− 732.73	7.34	2	**0.03**
Model 2	5	− 722.73	− 708.09	366.36	− 732.73			
Model 3	8	− 718.19	− 694.77	367.09	− 734.19	1.46	3	0.69
DV = swing phase								
Model 1	3	− 719.39	− 710.61	362.69	− 725.39			
Model 2	5	− 722.73	− 708.09	366.36	− 732.73	7.3402	2	**0.03**
Model 2	5	− 722.73	− 708.09	366.36	− 732.73			
Model 3	8	− 718.19	− 694.77	367.09	− 734.19	1.4615	3	0.69
DV = IA_min								
Model 1	3	575.54	584.32	− 284.77	569.54			
Model 2	5	579.02	593.66	− 284.51	569.02	0.5207	2	0.77
Model 2	5	579.02	593.66	− 284.51	569.02			
Model 3	8	579.16	602.57	− 281.58	563.16	5.8649	3	0.12
DV = IA_var								
Model 1	3	− 47.986	− 39.204	26.993	− 53.986			
Model 2	5	− 55.614	− 40.978	32.807	− 65.614	11.628	2	** < 0.01**
Model 2	5	− 55.614	− 40.978	32.807	− 65.614			
Model 3	8	− 53.058	− 29.640	34.529	− 69.058	3.4438	3	0.33

The random effect structure of participants was retained (random intercept, 1| ID). DV is the dependent variable and ID is the number of participants.

*DHI* Dizziness Handicap Inventory, *IA* inclination angle in the frontal plane, *Var* variance.

**Table 3:** Changes in clinical measurements during repeated tests in uVN.

	1st test	2nd test	3rd test	P-value	Post-Hoc
DHI	38.52 (24.94)	25.56 (20.26)	18.52 (19.69)	** < 0.01**	**1st ≠ 2nd, 1st ≠ 3rd**
Vestibular score	48.04 (25.69)	60.39 (20.50)	62.86 (16.95)	**0.02**	**1st ≠ 2nd, 1st ≠ 3rd**
Composite score	67.52 (12.45)	74.17 (7.78)	74.43 (8.57)	** < 0.01**	**1st ≠ 2nd, 1st ≠ 3rd**

1st test were conducted within 2 weeks of Department of Otorhinolaryngology-Head and Neck Surgery. 2nd test and 3rd test were conducted after 4 weeks and 8 weeks of 1st test. P-values are from the statistical test for the time effect on variables. Values are presented as mean (SD).

*uVN* unilateral vestibular neuritis, *DHI* Dizziness Handicap Inventory.

### Changes in temporo-spatial parameters

Compared to the null model, which had only random effects of participants, introducing a fixed effect (time) significantly improved the fit of the model for dependent variables, speed (chisq = 6.79, P = 0.03), stride length (chisq = 7.11, P = 0.03), step width (chisq = 6.25, P = 0.04), and proportion of stance phase (chisq = 7.34, P = 0.03) (Table 2). Introducing side effects did not improve the fit of the model for step length, proportion of the stance phase, or proportion of the swing phase. Post-hoc analysis showed significant differences between the 1st and 3rd tests in walking speed and stride length (Table 4). Step width showed a significant difference between the 1st and the other tests (2nd and 3rd tests) (Table 4).

**Table 4:** Changes in gait metrics during repeated tests for uVN and comparison between uVN and control.

	1st test	2nd test	3rd test	p-value	Post-Hoc	Control	t-test results
Speed (m/s)	1.09 (0.17)	1.14 (0.14)	1.16 (0.13)	**0.04**	**1st ≠ 3rd**	1.15 (0.12)	
Stride length (m)	1.16 (0.13)	1.20 (0.14)	1.22 (0.12)	**0.03**	**1st ≠ 3rd**	1.20 (0.07)	
Cadence (steps/min)	112.76 (13.74)	113.50 (6.46)	114.25 (8.17)	0.73		115.08 (9.24)	
Step width (m)	0.12 (0.04)	0.11 (0.03)	0.11 (0.03)	**0.05**	**1st ≠ 2nd , 1st ≠ 3rd**	0.10 (0.02)	**1st ≠ control ,**
Step length in affected(m)	0.59 (0.07)	0.60 (0.07)	0.61 (0.06)	0.23		0.60 (0.03)	
Step length in nonaffected (m)	0.58 (0.07)	0.60 (0.06)	0.61 (0.06)	0.12		0.60 (0.03)	
Stance phase in affected (%)	63.11 (2.17)	62.01 (1.80)	62.21 (1.82)	**0.01**	**1st ≠ 2nd , 1st ≠ 3rd**	62.14 (1.18)	
Stance phase in nonaffected (%)	62.58 (1.99)	62.00 (1.64)	62.27 (2.00)	0.26		62.14 (1.18)	
Swing phase in affected (%)	36.69 (2.17)	37.98 (1.80)	37.79 (1.82)	**0.01**	**1st ≠ 2nd , 1st ≠ 3rd**	37.85 (1.18)	
Swing phase in nonaffected (%)	37.42 (1.99)	38.00 (1.65)	37.73 (2.00)	0.26		37.85 (1.18)	
IA_min in affected (deg)	2.41 (2.33)	2.18 (1.95)	2.77 (1.00)	0.58		2.21 (0.53)	
IA_min in nonaffected (deg)	3.40 (2.25)	3.08 (2.30)	2.64 (1.06)	0.32		2.21 (0.53)	**1st ≠ control**
IA_var in affected (deg)	0.57 (0.19)	0.43 (0.17)	0.41 ( 0.13)	** < 0.01**	**1st ≠ 3rd**	0.44 (0.14)	**1st ≠ control**
IA_var in nonaffected (deg)	0.53 (0.28)	0.52 (0.23)	0.43 (0.13)	0.23		0.44 (0.14)	

1st test were conducted within 2 weeks of Department of Otorhinolaryngology-Head and Neck Surgery. 2nd test and 3rd test were conducted after 4 weeks and 8 weeks of 1st test. p-values are from the statistical test for the time effect on gait metrics. T-test results describe significantly different groups between control and uVN at each time. T-test was conducted for control and each time, respectively. Values are mean (SD).

*uVN* unilateral vestibular neuritis, *IA* inclination angle in frontal plane, *var* variability, *SS* single stance phase, *min* minimum.

### Changes in inclination angle

Compared to the null model, which had only random effects of participants, introducing a fixed effect (time) significantly improved the fit of the model for IA_var on the affected side (chisq = 11.63, P < 0.01) (Table 2). However, introducing a fixed effect (side) did not improve the fit of the model for IA_var. Post-hoc analysis showed a significant difference between the 1st and 3rd tests in IA_var on the affected side (Table 4).

### Comparison to controls

The exploration of gait parameters compared to the controls is reported in Table 4. The step width showed a significant difference from the controls in the 1st test. IA_var showed significant differences with controls in the 1st test on the affected side.

## Discussion

In the present study, perception of limitations in the activities of daily life after uVN were measured using the DHI^14^. The average DHI (38.52) in the 1st test corresponded to the results of previous studies for acute uVN (range 37–45)^21–23^. DHI improved significantly between the 1st and the 2nd tests. Although the DHI improved between the 2nd and 3rd tests, the difference was not statistically significant. We speculate that statistical insignificance between the 2nd and 3rd tests resulted from the large inter-personal variations. Considering DHI scores less than 5 in the previous study control group^23^, above findings suggest that the perception of limitation in the activities of daily life after uVN persists after 2 months of onset and support a previous study result that DHI improved to near normal values after 6 months^22^. The results of the vestibular score and composite score from the CDP showed significant improvements between the 1st and 2nd tests. Compared to the normal reference values^17^, the vestibular score and composite scores in the 2nd test in this study improved to the values within one SD of the normal reference values.

Gait characteristics with poor balance^12^ or perceptive dizziness^15^ are slow walking speed, short stride/step length, increased percentage of double support and stance phases, increased step width, and increased spatio-temporal variability. Previous investigations for vestibular disorders demonstrated gait characteristics different from healthy controls: slow walking speed, reduced cadence, and short step length^9,10^. This study focused on the longitudinal change of gait function after acute uVN. In this study, walking speed, stride length, and cadence did not show significant differences between participants with uVN and control in the 1st, 2nd, and 3rd tests, respectively. These findings suggest that the overall walking function represented by walking speed is within the normal range after the acute stage of uVN. However, speed and stride length showed significant improvement between the 1st and the 3rd test. These findings suggest a continuous improvement of overall walking function during the recovery stage (two weeks to three months). Participants with uVN showed wider step width in the 1st test than the controls. Step width improved significantly between the 1st and 2nd tests, thereby showing no significant difference from the controls in the 2nd test. Step width is related to the foot placement strategy in balance control by widening the base of support (BoS). The above results suggest that widening the step width is a temporary compensation strategy occurring during the early recovery stage. Participants with uVN did not show a significant difference in stance phase proportion compared to the control. Although stance phase proportion in the affected side significantly improved between the 1st and 2nd tests, it was within the normal range, even in the 1st test. Increased stance phase proportion is also related to balance control by reducing the swing phase which corresponds to the single support phase of the opposite side and is dynamically unstable. These findings also suggest that an increased stance phase proportion is a temporary compensation strategy during the recovery stage. From the gait metrics in the present study, widening of step width and increasing stance phase proportion may be compensation strategies observed in the early recovery stage of uVN. Even after these compensation strategies subsided, improvement of the overall walking function, represented by walking speed, continued during the recovery stage. Although spatio-temporal metrics are reliable, easy to take up, and most frequently studied, they have limitations in providing direct evidence of biomechanical or motor control and evidence of balance control.

Although CDP was thought as a useful test for monitoring the resolution of subjective symptoms in uVN^24^, most previous studies using CDP reported the CoM-CoP relationship only in the sagittal plane. However, the CoM-CoP relationship in the frontal plane might be more relevant to gait stability than in the sagittal plane^25^. Mediolateral stability during gait is maintained when CoM and extrapolated CoM are controlled within the BoS. In the previous studies, the maximum horizontal separation distance in frontal plane between CoM and CoP during stance phase was reported to sensitively quantify gait instability in patients with bilateral vestibular hypofunction or cerebellar ataxia^26,27^. The position of CoM close to the CoP was related to falls, and excessive lateral momentum of CoM was identified in the balance-impaired elderly^28^. We assume that the increased distance between CoM and CoP during the stance phase may result from a compensation related to the widening of the BoS.

In this study, IA_min in the non-affected side in the 1st test was significantly larger than that in the control. This finding may result from relative postural sway on the affected side and increased step width in uVN. Although there was a trend to decrease IA_min in the non-affected side, the time effect on this metric was not significant. We contemplate that this statistical insignificance results from large inter-personal variances. Therefore, future studies should be conducted to verify the results of the present study. There were no significant differences in IA_min between the control and the affected side of uVN in the 1st, 2nd, and 3rd tests. These results suggest maintenance of the relationship between CoM and CoP during the recovery stage of uVN, while the step width increases in the 1st test. This supports that the CoM-CoP relationship in the frontal plane is a dominant constraint for maintaining gait. In addition to the foot placement strategy, which is represented by step width, the ankle strategy may also influence the CoM-CoP relationship by medio-lateral shifting of CoP. Therefore, future studies should be conducted to reveal the influence of ankle strategy on uVN.

In this study, uVN showed significantly larger IA_var in the affected side in the 1st test compared to controls. Additionally, IA_var decreased significantly in the 3rd test on the affected side compared to that in the 1st test. We speculate that IA_var reflect the mediolateral balance control more directly than step width and proportion of stance phase because step width and proportion of stance phase are influenced by compensation strategies. Therefore, this finding indicates a continuous improvement of mediolateral balance control at least two months after uVN onset. Human motor performance is generated by an inherently ‘noisy’ nervous system, which results from stochastic events at the level of ion channels, synapses, neurons, and neural networks^29^. After uVN, noise in the nervous system increases, thereby increasing uncertainty and variability in vestibular nervous system. It is widely believed that motor control is optimized for current performance; the variability that interferes with this goal should be minimized^30^. However, variability in motor performance is a mean of exploring motor spaces that reinforce motor learning^31^. We think that the increased variability after the acute stage of uVN suggests the existence of an actively ongoing adaptation process in the vestibular system. Therefore, this period is clinically significant for long-term progress. Furthermore, more active rehabilitation should be provided because vestibular rehabilitation interventions interact with the recovery mechanism during the critical plastic time window of internal reorganization processes^2^.

This study has some limitations. First, gait metrics were not evaluated during the acute stage of uVN due to safety issues, making time differences between 3D gait analysis and other tests. Second, the duration for follow-up was too short to investigate the complete recovery of participants. Third, relationship between clinical tests for central compensation and gait recovery was not investigated. Fourth, the sample size was small for the t-test to compare the results of uVN and control. Larger sample size may be helpful for finding precise changes in gait parameters. Finally, although vestibular ocular rehabilitation exercise was provided at the acute stage of uVN, quantitative monitoring for this exercise program was not implemented. Future studies with longer follow-up periods, larger sample sizes, and control of rehabilitation programs should be conducted to verify the results of this study.

## Conclusions

Gait metrics representing overall gait function and mediolateral stability showed improvement during the recovery stage of uVN. Sequentially, step width and the proportion of stance phase, then walking speed, and variability of CoM-CoP relationship improved. These findings suggest that the improvement of dynamic stability during gait continues after two months of uVN onset, although walking speed and step width are within the normal range. We believe that clinicians should make efforts to provide vestibular rehabilitation for more than two months after uVN onset, thereby enhancing appropriate neural plasticity for dynamic stability during walking.

## Data Availability

The datasets generated or analyzed during the current study are available from the corresponding author upon reasonable request.

## Abbreviations

uVN: Unilateral vestibular neuritis
VOR: Vestibulo-ocular reflex
CoM: Center of mass
CoP: Center of pressure
DHI: Dizziness Handicap Inventory
CDP: Computerized dynamic posturography
SOT: Sensory organization test
IA: Inclination angle
IA_aff: IA in the affected side
IA_nonaff: IA in the non-affected side
IA_min: Minimum value of IA
IA_var: Variability of IA
LMM: Linear mixed model
BoS: Base of support
